# The development of precision medicine in clinical practice

**DOI:** 10.1186/s40169-015-0069-y

**Published:** 2015-08-25

**Authors:** Mingyan He, Jinglin Xia, Mohamed Shehab, Xiangdong Wang

**Affiliations:** Zhongshan Hospital, Minhang Hospital, Zhongshan Hospital Biomedical Research Center, Fudan University Medical School, Fudan University Center for Clinical Bioinformatics, Shanghai Institute of Clinical Bioinformatics, Shanghai, China; San Juan Bautista School of Medicine, San Juan, PR USA

**Keywords:** Precision medicine, Translational medicine, Data, Drug

## Abstract

Precision medicine allows a dramatic expansion of biological data, while there is still an urgent need to understand and insight the exact meaning of those data to human health and disease. This has led to an increasing wealth of data unanalyzed. The concept of precision medicine is about the customization of healthcare, with decisions and practices tailored to an individual patient based on their intrinsic biology in addition to clinical “signs and symptoms”. Construction of a standardized model for the integration of data from various platforms is the central mission of the ‘New Disease Management Model’. The model is helpful for the development of new taxonomy of diseases and subtypes, to personalize therapy based on patient genetic profiles. A rapid progression of precision therapy has been made recently. Clinical trials have shown the therapeutic efficacy of discovered and developed therapeutic agents has improved. However, next-generation drugs would be designed for disease subtypes with more specificity, efficacy and lower toxicity.

An enormous amount of biological data are generated and continues to expand dramatically, with the completion of Human Genome Project and the introduction of the next-generation sequencing. The efficiency of compiling, organizing and analyzing the data to extract a true understanding of fundamental biological processes of human health and disease has not kept pace, leading to an increasing wealth of unanalyzed data [1]. In 2011, national research council of the national academies have published “Toward precision medicine: Building a knowledge network for biomedical research and a new taxonomy of disease”, which suggests that genomic findings promote the integration of information between biomedical research and clinical medicine [1].

Precision medicine aims to harness large amounts of data available from the human genome and the subsequent research wave of the molecular basis of disease. This allows the customization of healthcare and integration of electronic medical information on both an individual and larger global scale. With decisions and practices being tailored to the individual patient based their intrinsic biology in addition to traditional physical signs and symptoms [2]. There is a distinct from other similar ideas of personalized medicine, in that precision medicine implies that unique treatments can be designed for each individual, severe taxonomic needs of the biomedical research and medical communities, providing a profound definition of diseases subtypes at the molecular level. Ultimately, precision medicine allows for more precise diagnosis and treatment at the intrinsic molecular level.

With advances of information technology, such as electronic medical records, it is possible to acquire detailed clinical data about individual patients and computational tools for analyzing large sets of data provide a means to search for unexpected correlations with enormous data. Recent development of large-scale and multilevel omics-based biotechnologies, e.g. proteomics, metabolomics, genomics, phenomics, diverse cellular assays, provides precision medicine with powerful methodological bases and creates great potentials for improving healthcare [3]. Precision oncology is referred to as the vanguard of precision medicine. Cancer is a genomic disease with a series of mutated or altered oncogenes, while oncology research has benefited from the large scale biotechnology studies of cancer genomes. The translation of analyzed data has also revealed scores of cancer genes affecting cell signaling, chromatin, epigenomic regulation, metabolism, and lineage maturation [4]. Recently, the Obama administration announced a research initiative geared towards precision medicine and to the new era of medicine both delivering the right treatment at the right time [5].

Translational medicine research is the main component of precision medicine’s integration of large sets of bioinformatics data with medical practice. Numerous translational research centers are emerging constantly and launch translational projects. In 2012, the International Personal Genome Project published an integrated map of genetic variation from 1092 human genomes, which revealed substantial genetic variation among populations. These variations will be helpful to understand genetic variations among different ethnic and racial backgrounds as well as how these variants affect drug metabolism [6, 7]. In 2013, British Prime Minister David Cameron, launched the ‘100,000 people genome project’, with results published and available to the public at no cost [8]. This genetic and phenotypic information revealed through this projection will enable researchers to use the data to identify genes linked to medical conditions, develop new treatments and facilitate improvements in precision medicine.

In addition, other large-scale projects, such as The Cancer Genome Atlas (TCGA), Encyclopedia of DNA Elements (ENCODE), or Human Proteome Project (HPP), were launched [9, 10]. TCGA contains somatic mutations, copy number variation, mRNA expression, miRNA expression, protein expression, and histology slides, and aims to develop an integrated landscape of complex molecular network for approximately 7000 human tumors. The ENCODE project has mapped regions of transcription, transcription factor association, histone modification, DNA methylation and chromatin structure to delineate all functional elements encoded in the human genome [11]. HPP aims to reach a detailed understanding of the ~20,000 human protein-coding gene predicted from the human genome. Recently, the Chromosome-centric Human Proteome Project [10, 12] and tissue-based maps of human proteomes [13] have been described. The project has extensively and systematically analyzed all missing and known proteins in chromosomes for their tissue/cellular/subcellular localizations as well as their associations with human diseases (i.e., immune diseases, metabolic disorders, and cancers). Tissue-based maps of human proteomes have identified differences in druggable proteins, cancer proteins, and their metabolism between different tissues and organs.

A secretome as complete sets of secreted proteins is an important part of the proteome. Secretomes encode approximately 10 % of the human genome and play important roles in cellular immunity, adhesion, communication, and various drug delivery mechanisms [14]. Analyses of secretomes have been reported in various organisms and cell types [15]. The Secreted Protein Database is a collection of over 18,000 secreted proteins from Human, Mouse and Rat proteomes, which includes sequences from SwissProt, Trembl, Ensembl and Refseq [16]. Studying cell secretomes can be helpful in identifying biomarkers needed for diagnosis, prognosis and/or monitoring therapeutic response [14].

There still remains the need to build a linked library network as well as new taxonomy profiles of diseases for the better implementation of precision medicine, although enormous data have been generated and shown contribution to precision diagnosis, pharmaceutical development. The network should be integrated for the convenience of searching information including individual genome, transcriptome, proteome, metabolome, phenome, data from clinical signs and symptoms, experimental tests, environmental exposures, and socioeconomic factors. This provides a deeper understanding of disease mechanisms, pathogenesis, treatment, and drive forward the development of new taxonomy to define disease subtypes. However, data from different platforms are not standardized. Building a standardized model for the integration of data is a major challenge.

Bioinformatics data provide great insights to the understanding of the biological processes of human diseases and health. Computational data mining methods are important tools for the prioritization of disease candidate gene. The gene network-based guilt-by-association is the basic principle from which genes with the same interaction partners or expression data, most likely share the same biological function [17]. There are various data sources and network measures for disease gene prioritization [18]. For example, Gene Ontology and KEGG enable the functional interpretation of genes and gene products via enrichment analysis. NCBI LocusLink, Ensemble, SwissPro, or TrEmbl provide protein sequence data, probably associated with diseases. Network analysis can be done using either local network information such as direct neighbor, shortest path or degree of a node. Global network information can be taken into account the entire network topology to rank candidate genes [18]. The limitation should not go unnoticed and under-developed, although data mining tools have greatly benefited researchers to identify candidate genes. For instance, not all functional data has been annotated in Gene Ontology and KEGG, resulting in inevitable omissions of potential key molecular pathways. Most of the mining methods rely on previous reported data information available for diseases. This limits the prediction potential for genes related to diseases.

The development of target-specific drugs has made great progression in improving therapeutic response of various diseases, on basis of a deeper biological understanding of diseases and an efficient validation of clinical therapeutic effects. For example, the epidermal growth factor receptor (EGFR) pathway has been targeted for the treatment of patients with somatic EGFR mutant non-small-cell lung carcinoma (NSCLC). EGFR tyrosine kinase inhibitors (TKIs) such as erlotinib and gefitinib have confirmed significant improvements in the ‘progression-free survival’, in advanced EGFR mutated NSCLC [19, 20]. Other target-specific drugs include HER2 targeted agents (e.g. lapatinib, pertuzumab), ABL inhibitors (e.g. imatinib, nilotinib), MEK inhibitor (e.g. trametinib), or immune checkpoint inhibitors (e.g. anti-CTLA4 monoclonal antibody agents, ipilimumab). Most of the therapeutic targets are critical molecular signaling pathways associated with development of disease. Targeting specific molecular or effectors of different diseases have shown therapeutic advantages and great potential as an important aspect of precision medicine.

However, a large number of challenges should be considered and overcome during the development and clinical application of targeted agents. Various toxicities and developments of new targeted molecular therapies were revealed [21], including cardiac, pulmonary, skin, endocrine, gastrointestinal toxicities caused by new targeted therapies. It is urgent to develop more specific, efficacious targeted therapies with low toxicities. Additionally, drug resistance has been inevitably observed in some patients who were expected to have a better response before use. The resistance mechanisms should be further investigated for potential solutions or the development of anti-resistant next-generation targeted agents. For example, the development of acquired EGFR inhibitor resistance have been studied to be involved in a number of mechanisms, such as the T790M missense mutation in exon 20 of the EGFR kinase domain and escape mechanisms through critical genetic drivers, including c-MET/HGF, HER2, PIK3CA, ERK, BRAF, CRKL, and AXL [22]. The next-generation EGFR inhibitors, such as afatinib, can bind to both EGFR and HER2 through covalent bonds resulting in the irreversible blockade, and be developed with more sustained target modulation [23]. The third-generation EGFR inhibitors to focus on T790M-mediated drug resistance are currently in clinical trial testing [23].

In conclusion, precision medicine has shown great potentialities while catering to the requirements of bioinformatics and medical practice development. Building a standardized model for the integration of data from various platforms is the central mission which is helpful for the development of new taxonomy of disease subtypes and personalized therapy based on patient genetic profiles. Furthermore, there has been progress towards the discovery and development of targeted therapeutic agents with evidence in clinical trials of the improvement of therapeutic efficacy. Next-generation drugs would be designed for diseases subtypes with more specificity, efficacy and lower toxicity.
